# Structure of the Essential *Plasmodium* Host Cell Traversal Protein SPECT1

**DOI:** 10.1371/journal.pone.0114685

**Published:** 2014-12-05

**Authors:** Brent Y. Hamaoka, Partho Ghosh

**Affiliations:** Department of Chemistry & Biochemistry, University of California San Diego, La Jolla, California, United States of America; Johns Hopkins Bloomberg School of Public Health, United States of America

## Abstract

Host cell traversal by *Plasmodium*, the protozoan cause of malaria, is an essential part of this parasite's virulence. In this process, the parasite enters a host cell through a parasite-induced pore, traverses the host cell, and then exits the host cell. Two *P. berghei* proteins, SPECT1 and SPECT2, are required for host cell traversal by the sporozoite form of the parasite. In the absence of either, no pore formation is observed. While SPECT2 has sequence homology to pore-forming proteins, SPECT1 has no homology to proteins of known structure or function. Here we present the 2.75 Å resolution structure of a slightly truncated version of *P. berghei* SPECT1. The structure reveals that the protein forms a four-helix bundle, with the rare feature of having all of these helices in parallel or antiparallel alignment. Also notable is the presence of a large, conserved, hydrophobic internal cavity in the protein, which may constitute a ligand-binding site or be indicative of partial instability in SPECT1, or both. The structure of SPECT1 will make possible targeted mutagenesis experiments aimed at understanding its mechanism of action in host cell traversal.

## Introduction

Malaria exerts a global burden on human health and welfare, being endemic in 100 countries and causing 250 million clinical illnesses and 1 million deaths each year [1], [2]. This devastating disease is caused by the infective sporozoite form of the protozoan parasite *Plasmodium*, which is introduced into the dermis of a host through the bite of an infected female Anopheline mosquito. Sporozoites are highly motile and transit from the dermis through the circulatory system to the liver, where they invade hepatocytes and mature into the red blood cell-infective form of the parasite [3], [4]. Prior to invading hepatocytes, sporozoites have the ability to traverse host cells [5]–[7]. Host cell traversal involves entry into a host cell by the sporozoite, transit through the host cell cytosol, and finally exit from the host cell. Entry and presumably exit require the formation of a pore in the host cell plasma membrane [5], [7], [8], resulting in a membrane wound that causes necrosis in most instances, although in the some cases the wound is resealed and the cell survives [5], [9], [10]. Host cell traversal was first observed in macrophages [11], but has since been documented in other cell types, including hepatocytes, epithelial cells, and fibroblasts [5], [7], [10]. There appears to be no cell type specificity for host cell traversal, suggesting that only *Plasmodium* factors are important for this process.

Host cell traversal plays a critical role in *Plasmodium* infectivity. Loss of host cell traversal results in a loss in infectivity due to the killing of the parasite by Kupffer cells, the resident macrophage of the liver [6], [8], [12]. A further loss in infectivity occurs due to the entrapment of sporozoites by phagocytic leukocytes as well as nonphagocytic cells in the dermis [7]. The combined loss in infectivity due to effects in the dermis and liver appears to be ∼300-fold (10-fold in the dermis and 30-fold in the liver).

Two proteins from *P. berghei*, a rodent-infecting parasite, have been identified to be essential for host cell traversal by sporozoites [8], [12]. These are *Pb*SPECT1 and *Pb*SPECT2 (sporozoite microneme protein essential for cell traversal). No pore formation or host cell membrane wounding is seen in the absence of either of these proteins [7], [8], [12]. This suggests that *Pb*SPECT1 and *Pb*SPECT2 work together or in consecutive fashion to form a pore. Both *Pb*SPECT1 and *Pb*SPECT2 have signal sequences and localize to micronemes [8], [12], secretory organelles at the apical end of sporozoites.

The sequence of *Pb*SPECT2 (also called *Plasmodium* perforin-like protein1, PPLP1) suggests that it has a direct role in pore formation. This protein of ∼800 amino acids has a central domain of ∼330 amino acids that has homology to pore-forming proteins, namely those belonging to the membrane attack complex/perforin (MACPF) and cholesterol-dependent cytolysin (CDC) family [12]–[15]. MACPF/CDC proteins are synthesized as soluble proteins and are triggered to undergo a conformational change that promotes insertion and pore formation in target membranes. Recombinant *P. falciparum* SPECT2 has been shown to cause lysis of red blood cells in a Ca^2+^-dependent manner, as has the MACPF/CDC domain of *Pf*SPECT2 [16]. The role of *Pf*SPECT2 in host cell traversal by sporozoites, however, has not been demonstrated. Instead *Pf*SPECT2 has been implicated in the Ca^2+^-dependent egress of *P. falciparum* merozoites from red blood cells [16]. *Pf*SPECT2 and *Pb*SPECT2 appear to have different functions, as the latter has been detected only in sporozoites and is absent from merozoites [12]. *Pb*SPECT2 has not yet been demonstrated to form a pore, and as noted above, the genetic data indicate that the presumed pore-forming activity of *Pb*SPECT2 is dependent on *Pb*SPECT1.

In contrast to *Pb*SPECT2, *Pb*SPECT1 (∼25 kDa) has no detectable primary sequence homology to proteins of known structure or function [8]. *Pb*SPECT1 has no identifiable membrane-spanning or attachment regions. *Pb*SPECT1 has been found to be expressed in salivary gland sporozoites by not in merozoites, ookinetes, or midgut sporozoites [8]. Homologs of *Pb*SPECT1 are encoded in the genomes of a number of *Plasmodium* species, including *P. falciparum*. These proteins have ∼40% sequence identity, suggesting that they share a common structure and mode of action. Homologs of SPECT1 do not exist outside of *Plasmodium* species. To begin to understand how *Pb*SPECT1 contributes to host cell traversal, we recombinantly expressed, purified, and determined the crystal structure of a slightly truncated version of this protein. We found that *Pb*SPECT1 forms a four-helix bundle, with the rare feature of having all of these helices in parallel or antiparallel alignment. This along with a large, conserved, hydrophobic internal cavity in *Pb*SPECT1 are suggestive of conformational lability in the molecule, which would accord with a mechanism in which the protein is triggered to undergo a conformational change from soluble to membrane-associated form, as typical for many pore-forming proteins (including those belonging to the MACPF/CDC family).

## Materials and Methods

### *P. berghei* SPECT1 expression and purification

*P. berghei* SPECT1Δ24, containing amino acids 25–241, and SPECT1Δ41, containing amino acids 42–241, were cloned from a plasmid generously provided by Masao Yuda into a modified pET28 vector (Novagen) as a C-terminal fusion to *S. cerevisiae* SUMO (SMT3, NP_010798.1); these constructs included an N-terminal His-tag preceding SUMO (Table S1). The expression vectors were separately transformed into *E. coli* BL21 (DE3). Bacteria were grown with shaking at 37°C in LB media containing 50 µg/mL kanamycin to an OD_600_ of 0.8–1.0, whereupon expression was induced with 0.4 mM IPTG; the cultures were maintained thereafter for 16–18 h at room temperature. Bacteria were harvested by centrifugation (2246×g, 20 min, 4°C), resuspended in lysis buffer (100 mM sodium phosphate buffer [Na*Pi*], pH 8.0, 500 mM NaCl, and 40 mM imidazole), and lysed using an Emulsiflex (Avestin). The lysates were clarified by centrifugation (37,500×g, 30 min, 4°C), filtered through a 0.45 µm filter, and applied to columns containing Ni^2+^-nitrilotriacetic acid (NTA, Sigma) agarose beads (10 mL per L of bacterial culture); prior to application, the columns were equilibrated in binding buffer (20 mM Na*Pi*, pH 8.0, 500 mM NaCl) containing 40 mM imidazole. The columns were washed with 10 column volumes of binding buffer, and bound proteins were eluted in binding buffer containing 250 mM imidazole.

The His-tag and SUMO fusion partners were cleaved from *Pb*SPECT1Δ24 and *Pb*SPECT1Δ41 using His-tagged *S. cerevisiae* ULP-1. The cleavage reaction was carried out overnight at a 4∶1 substrate∶ULP-1 molar ratio at 4°C in binding buffer supplemented with 250 mM imidazole, 1 mM DTT, and 5 mM EDTA. The samples were then dialyzed (6000–8000 MWCO membrane) in binding buffer at 4°C and applied to Ni-NTA columns in the same buffer. Fractions of *Pb*SPECT1Δ24 or *Pb*SPECT1Δ41 that were devoid of their fusion partners were collected from the flow-through of these columns. These fractions were then exchanged into 20 mM Tris, pH 8, 50 mM NaCl using a HiPrep 26/10 desalting column (GE Healthcare) and applied in this same buffer to a Q Sepharose anion exchange column (GE Healthcare). Protein that was bound to this column was eluted using a step-wise gradient to 20 mM Tris pH 8, 200 mM NaCl. *Pb*SPECT1Δ24 and *Pb*SPECT1Δ41 were further purified by gel filtration chromatography (Superdex 75 26/60, GE Healthcare) in 20 mM Tris, pH 8, 10 mM NaCl. Purified *Pb*SPECT1Δ24 and *Pb*SPECT1Δ41 were concentrated by binding the proteins to a 1 mL HiTrap Q anion exchange column (GE Healthcare) and eluting the protein using a step-wise gradient to 20 mM Tris, pH 8, 500 mM NaCl. The concentrated proteins were dialyzed at 4°C using a 3500 MWCO Slide-A-Lyzer (Thermo Scientific) in 1 mM Tris, pH 8, 10 mM NaCl. The dialyzed proteins were flash frozen in liquid N_2_ and stored at −80°C.

Two dual Met-substitution mutants of *Pb*SPECT1Δ41, L57M/L75M and L57M/I133M, were constructed for the purpose of phasing. The mutagenesis was carried out using a combination of MegaPrimer [17] and QuikChange (Agilent Technologies, Inc.) techniques (Table S1). Selenomethionine (SeMet) was incorporated into *Pb*SPECT1Δ41 L57M/I133M using methionine pathway inhibition as described previously [18], except that biotin was omitted and 20% glucose (w/v) was included in the M9 minimal salts media. SeMet-labeled SPECT1Δ41 L57M/I133M was expressed and purified as described above. *Pb*SPECT1Δ41 L57M/L75M was produced without SeMet-labeling, and expressed and purified as described above; this protein was derivatized with thimerosal, as described below, for the purpose of phasing.

### Proteinase K Digestion

*Pb*SPECT1Δ24 (4 µg) was incubated with 3×10^−5^ µg, 3×10^−6^ µg, 3×10^−7^ µg, 3×10^−8^ µg, or 3×10^−9^ µg proteinase K in a 50 µL reaction volume containing 25 mM Tris, pH 8.0, 25 mM CaCl_2_, 75 mM NaCl for 1 hr at 37°C. Digestion products were visualized by Coomassie-stained reducing SDS-PAGE.

### Multiangle Light Scattering

Absolute molecular masses were determined by multiangle static light scattering. Protein samples in 150 mM NaCl, 50 mM Tris or HEPES, pH 7.4 were applied to a TSK-Gel G3000 SWxl size-exclusion column (Tosoh, Bioscience) attached to a miniDAWN TREOS SLS detector and an Optilab T-rEX refractive index detector (Wyatt, Santa Barbara, CA). Data were processed using ASTRA 5 software (Wyatt, Santa Barbara, CA).

### *C*rystallization

*Pb*SPECT1Δ24 was crystallized by hanging drop vapor diffusion. The wells of a 24-well plate were filled with 1 mL of 0.1 M Bis-tris methane, pH 5.7, 2.5% (w/v) PEG 400, 1.2 M (NH_4_)_2_SO_4_. *Pb*SPECT1Δ24 at ∼13 mg/mL was mixed with the reservoir buffer at a 1∶1 ratio in a 1 µL crystallization drop.

Native *Pb*SPECT1Δ41 was crystallized using an Oryx8 Crystallization Robot (Douglas Instruments) in a 96-well sitting drop plate (CrystalClear Duo, Douglas Instruments). The reservoir contained 0.08 mL of 0.2 M MgCl_2_•6H_2_O, 0.1 M HEPES, pH 7.5, and 25% (w/v) PEG 3350. Crystallization drops were 1 µL and contained 70% protein at 19.3 mg/mL (determined using an ε_280_ of 8300 M^−1^cm^−1^) and 30% reservoir solution. Plates were incubated at 18°C. Clusters of crystals formed after ∼2 weeks. The clusters were mechanically broken up and large fragments were dragged using a loop through an oil mixture consisting of 3∶1 paraffin∶Paratone N before flash cooling in liquid N_2_. X-ray diffraction data were collected at beamline 23-IDB at APS.

SeMet-labeled *Pb*SPECT1Δ41 L57M/L133M was crystallized by a modified batch method. The wells of a 24-well dish were filled with 0.5 mL of 0.3–0.4 M MgCl_2_•6H_2_O, 0.1 M HEPES, pH 7.5, and 30–37% (w/v) PEG 3350. SeMet-labeled *Pb*SPECT1Δ41 L57M/L133M at ∼45 mg/mL was mixed with the reservoir buffer at a 1∶1 ratio in a 2 µL crystallization drop. The reservoir buffer was diluted 1∶1 with water before sealing the well with a coverslip. Crystals formed within ∼4 days and were cryopreserved as described above for native crystals.

*Pb*SPECT1Δ41 L57M/L75M was crystallized by the modified batch method described above. Mercury derivatization of crystals of this protein was achieved by picking up a single crystal of thimerosal using a loop and adding it directly into the crystallization drop containing the protein crystal; the thimerosal crystal was observed to dissolve under a microscope. The *Pb*SPECT1Δ41 L57M/L75M crystal was incubated in thimerosal at 18°C for 2 hours before flash cooling as described above.

### Structure Determination

Native diffraction data were collected at APS beamline 23-ID-D from a crystal of *Pb*SPECT1Δ41, which belonged to space group P2_1_2_1_2_1_ and had four *Pb*SPECT1Δ41 molecules in the asymmetric unit. Multiple wavelength anomalous dispersion (MAD) data were collected at SSRL beamline 9.2 from a crystal of SeMet-labeled *Pb*SPECT1Δ41 L57M/L133M, and single wavelength anomalous dispersion (SAD) data were collected at APS beamline 23-ID-B from a crystal of thimerosal-derivatized *Pb*SPECT1Δ41 L57M/L75M. Crystals of Met-substituted *Pb*SPECT1Δ41 belonged to space group C222_1_, and had two *Pb*SPECT1Δ41 molecules in the asymmetric unit. Crystallographic data were processed with HKL2000 [19]. The SeMet MAD and thimerosal SAD datasets were assigned separate phasing groups in Phenix's AutoSol program [20] and the phasing information from both datasets were combined into a single set of phases. Four selenium sites and two mercury sites were identified in each asymmetric unit, and the density modified electron density map showed clear evidence for a four-helix bundle. Four polyalanine α-helices were manually placed into electron density maps using Coot [21]. The resulting model was used in Phenix as a search model in molecular replacement phasing of diffraction data from crystals of native *Pb*SPECT1Δ41 [20]. Electron density for amino acid side chains became more apparent in the resulting electron density map, and side chains were assigned based on the positions of the anomalously scattering atoms. Twenty-four iterations of maximum likelihood restrained refinement using Phenix (with default parameters) followed by manual rebuilding into σ_A_-weighted 2mFo-DFc and mFo-DFc maps using COOT were carried out. Non-crystallographic symmetry restraints were applied at each step of refinement using default parameters in Phenix. Electron density for the main chain was continuous for the following residues: 48–82, 88–197, and 206–240 in chain A; 50–82, 89–239 in chain B; 47–82, 86–167, 174–197, and 210–239 in chain C; and 47–82, 91–197, and 207–240 in chain D. Analysis by MolProbity [20] yielded a clashscore of 5.8 and a MolProbity score of 1.74; MolProbity reports these as the 100^th^ percentile for both score categories.

Molecular figures were made with PyMol (). Structure-based sequence alignments were generated using 3D-Coffee [22] and displayed using ESPript [23]. Mapping of conserved residues to the molecular surface of *Pb*SPECT1Δ41 was carried out with ConSurf [24]. Analysis of the cavity in *Pb*SPECT1Δ41 was carried out with CASTp [25]. The crystal structure and structure factors have been deposited with the Protein Data Bank (accession code 4U5A).

## Results and Discussion

**Figure 1:**
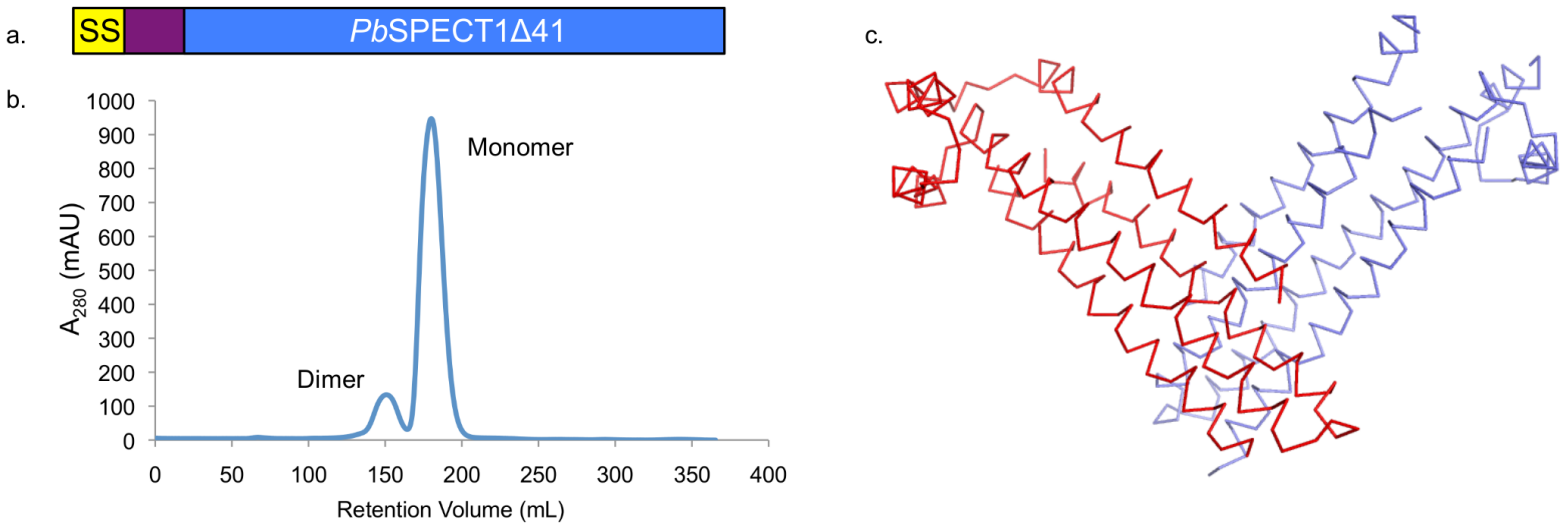
Monomeric and Dimeric Forms of *Pb*SPECT1Δ41. **a**. Schematic to scale of *Pb*SPECT1. Yellow denotes the putative signal sequence, purple the portion of the mature protein not included in *Pb*SPECT1Δ41, and blue *Pb*SPECT1Δ41. **b**. Size exclusion chromatogram of *Pb*SPECT1Δ41 showing the appearance of monomeric and dimeric species. **c**. “V”-shaped association between two *Pb*SPECT1Δ41 molecules (red and blue) observed in the asymmetric unit of the crystal.

We sought to crystallize and determine the structure of *Pb*SPECT1 to gain insight into its mechanism of action in host cell traversal. A slightly truncated version of *Pb*SPECT1 containing amino acids 25–241 (called *Pb*SPECT1Δ24) was initially chosen for crystallization trials. The first nineteen amino acids of *Pb*SPECT1 make up a predicted signal sequence [26] and were therefore excluded from this construct (Fig. 1a), and the first several amino acids of mature *Pb*SPECT1 are predicted to be disordered [27] and were therefore also excluded from this construct. *Pb*SPECT1Δ24 was expressed in *E. coli*, purified, and crystallized. These crystals, however, were found to yield no observable X-ray diffraction (with the use of a synchrotron source) under a variety of cryoprotectant conditions. Similarly, no diffraction was observed (using a rotating anode source of X-rays) when these crystals were held at room temperature.

To overcome this issue, a further truncation of *Pb*SPECT1 was carried out. For this, *Pb*SPECT1Δ25 was subjected to proteinase K digestion, resulting in the production of a stable, shorter fragment. This fragment consisted of residues 42–241, as identified by N-terminal sequencing and MALDI-TOF mass spectrometry. A construct encoding *Pb*SPECT1 residues 42–241, called *Pb*SPECT1Δ41 (Fig. 1a), was then expressed in *E. coli* and purified. *Pb*SPECT1Δ41 was observed by size-exclusion chromatography (SEC) to exist as two separable species (Fig. 1b). These species were stable, as the isolated forms maintained their state over the course of days as assessed by SEC. Multiangle light scattering, which provides a shape-independent measure of mass, showed that these species corresponded to monomeric (M_n_ 21,380 Da; calculated 23,184 Da) and dimeric (M_n_ 45,070 Da) forms of SPECT1Δ41 (Fig. S1). While the dimeric form of *Pb*SPECT1Δ41 did not crystallize, the monomeric form did. It is worth noting that *Pb*SPECT1Δ24 also formed monomeric and dimeric species, and it was the monomeric form that yielded the non-diffracting crystals mentioned above.

Crystals of *Pb*SPECT1Δ41 diffracted to 2.7 Å resolution (Table 1), and its structure was determined by a combination of isomorphous replacement and multi-wavelength anomalous dispersion (Fig. S2). While monomeric *Pb*SPECT1Δ41 was used in the crystallization, an association suggestive of a dimer was seen in the asymmetric unit. Two *Pb*SPECT1Δ41 molecules came together in a “V”-shaped arrangement, with a total of ∼1260 Å^2^ being buried at the interface (Figs. 1c and S3). This is the largest intermolecular buried surface area in the crystal, with the next largest being ∼1020 Å^2^. The asymmetric unit is constituted by two of these “V”-shaped, presumptive dimers that are then related by noncrystallographic translational symmetry (Fig. S3b). In crystals of methionine-substitution mutants of *Pb*SPECT1Δ41 (produced for phasing purposes), the same intermolecular packing was seen but the translational noncrystallographic symmetry became crystallographic (changing the space group from P2_1_2_1_2_1_ to C222_1_). Whether *Pb*SPECT1 forms oligomers in effecting host cell traversal is not known, but the dimeric state observed by SEC and the association observed in the crystal raise the possibility that a dimer may be involved.

**Table 1:** Crystallographic Data Collection and Refinement.

	*Pb*SPECT1Δ41	Thimerosal-derivatized *Pb*SPECT1Δ41 L57M/L75M	SeMet *Pb*SPECT1Δ41 L57M/L133M (edge)	SeMet *Pb*SPECT1Δ41 L57M/L133M (peak)	SeMet *Pb*SPECT1Δ41 L57M/L133M (remote)
Wavelength (Å)	1.00726	1.00800	0.97925	0.97908	0.96109
**Data collection**					
Space group	P 2_1_ 2_1_ 2_1_	C 2 2 2_1_	C 2 2 2_1_	C 2 2 2_1_	C 2 2 2_1_
Cell dimensions					
* a*, *b*, *c* (Å)	55.2 64.3 233.7	56.2 64.0 236.2	55.8 64.1 239.4	55.8 64.1 239.4	55.8 64.1 239.4
α, β, γ (°)	90 90 90	90 90 90	90 90 90	90 90 90	90 90 90
Resolution (Å)	49.90–2.75 (2.85–2.75)*	50.00–3.50 (3.56–3.50)	50.00–3.52 (3.65–3.52)	50.00–3.51 (3.64–3.51)	50.00–3.45 (3.57–3.45)
*R*_merge_	0.134 (0.618)	0.055 (0.140)	0.055 (0.160)	0.056 (0.165)	0.053 (0.170)
*I/*σ*I*	9.61 (2.46)	26.5 (11.5)	21.3 (5.2)	21.0 (4.9)	19.2 (5.2)
Completeness (%)	98.6 (87.8)	99.3 (100.0)	83.7 (36.0)	83.4 (33.8)	81.2 (32.3)
Redundancy	11.3 (4.6)	4.7 (4.6)	5.1 (3.1)	5.1 (3.0)	5.0 (2.9)
Mean Figure of Merit		0.331	0.510		
**Refinement**					
Resolution (Å)	49.90–2.75 (2.85–2.75)*				
No. reflections	22242 (1924)				
*R*_work_/*R*_free_	0.243(0.323)/0.271 (0.339)				
No. atoms	5588				
Wilson *B*-factor (Å^2^)	52.5				
Average *B*-factor (Å^2^)	48.0				
R.m.s. deviations					
Bond lengths (Å)	0.004				
Bond angles (°)	0.84				
Ramachandran Statistics					
Favored	95.65%				
Allowed	4.06%				
Outliers	0.29%				

**Figure 2:**
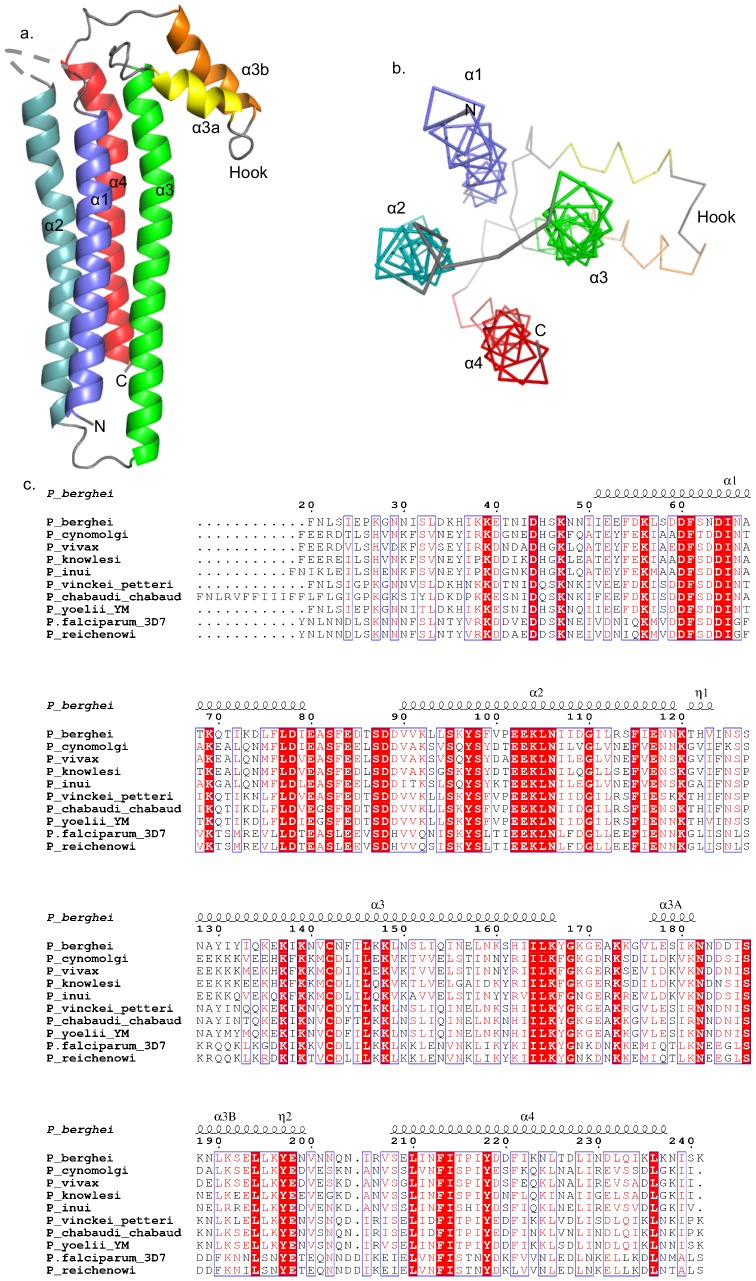
Structure and Conservation of *Pb*SPECT1Δ41. **a**. Ribbon representation of the four-helix bundle structure of *Pb*SPECT1Δ41 (4U5A), with individual α-helices in different colors. **b**. The structure of *Pb*SPECT1Δ41 viewed down the axis of the four-helix bundle. Coloring of individual α-helices is as in panel a. **c**. Structure-based sequence alignment of *Pb*SPECT1Δ41 (Genbank BAD08209.1, PlasmoDB PBANKA_135560) with *Plasmodium* homologs: *P. cynomolgi* (XP_004223591.1, PCYB_122110), *P. vivax* (PVX_083025), *P. knowlesi* (CAQ41197.1, PKH_121200), *P. inui* (EUD67722.1), *P. vinckei petteri* (EUD71736.1), *P. chabaudi chabaud* (PCHAS_136020), *P. yoelii* YM (PYYM_1357700), *P. falciparum* 3D7 (PF3D7_1342500), and *P. reichenowi* (CDO66209.1). Putative secretion signals were excluded. The secondary structure of *Pb*SPECT1Δ41 is shown above the sequence. Absolutely conserved residues are in white on a red background, and similar residues are in red; both types are in blue boxes.

The structure of *Pb*SPECT1Δ41 revealed that it forms a four α-helix bundle with a ‘hook’-like feature at one end (Figs. 2a and 2b). No electron density was visible for the first 5–9 residues or the last 1–2 residues of *Pb*SPECT1Δ41, likely due to the flexibility of these amino acids. The helices in the four-helix bundle range between 29–39 residues in length and proceed in an up-and-down fashion. Helices 2 (α2) and 4 (α4), which pack against one another, contain prolines (P101 in α2 and P216 in α4) and are kinked due to these. These prolines are not conserved in *Pb*SPECT1 homologs (Fig. 2c), and thus the kinks are unlikely to be functionally consequential. The connections between the four helices in the bundle are between nearest neighbors, except for a diagonal connection between α2 and α3. These connections mainly consist of short loops. The loop connecting α1 with α2 appears to be highly flexible, as no density for 4–8 residues in this loop, depending on the *Pb*SPECT1Δ41 molecule, was evident. Analysis of crystals confirmed that the lack of density in this region was not due to inadvertent proteolysis in the loop (Fig. S4). The “hook” feature of *Pb*SPECT1Δ41 is composed of short antiparallel α-helices (α3a and α3b) that are situated obliquely to the four-helix bundle and form a connection between helices α3 with α4. The segment leading from the “hook” to the α4 helix appears also to be flexible, as electron density for this region (residues 198–205) was visible in only one of the four *Pb*SPECT1Δ41 molecules in the asymmetric unit; this electron density was sufficient for modeling only the main chain. Other than this difference, the four SPECT1Δ41 molecules are nearly identical in structure (root-mean-square-deviation ranging between 0.49–0.87 Å for main chain atoms).

**Figure 3:**
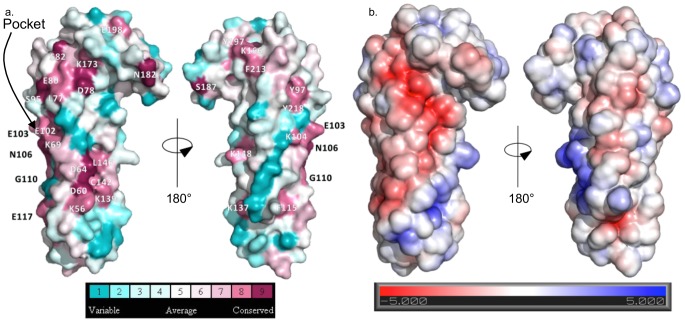
Surface of *Pb*SPECT1Δ41. **a**. Residues conserved among homologs of *Pb*SPECT1 were mapped to the molecular surface of *Pb*SPECT1Δ41. Two views of *Pb*SPECT1 related by a 180° rotation around the indicated axis are shown. Color scale for conservation is shown at bottom. **b**. The electrostatic surface mapped to the van der Waals surface of *Pb*SPECT1Δ41 is shown. As in panel a, two views and a color scale are shown.

One face of *Pb*SPECT1Δ41 has several patches of conserved residues (Fig. 3a, left). One of these patches (composed of L77, D78, E80, S82, S95, K173) is proximal to the “hook” and has an overall negatively charged electrostatic character (Fig. 3b, left). The other (K56, D60, D64, K139, C142, L146) is distal to the “hook” and has a negatively charged stripe separated from a positively charged one. These two patches are connected by a set of conserved residues that line a deep pocket (K69 and E102, among others) that is described below. The opposite face of *Pb*SPECT1Δ41 is more variable and has no distinctive electrostatic character (Figs. 3a and 3b, right).

The four-helix bundle is a common structural motif and occurs in proteins of diverse functions [28]. For the vast majority of four-helix bundle proteins, the helices are tilted at about +25° or -35° with respect to one another [28]–[31]. This optimizes the packing between side chains. In contrast to this pattern, the helices in *Pb*SPECT1Δ41 are nearly parallel or antiparallel to one another; the angles between the helices range between 1–8° from parallel/antiparallel. Parallel/antiparallel packing is rare in four-helix bundle proteins, but has been observed to occur at a frequency greater than that predicted by random chance [30], [31]. This latter point suggests that the parallel/antiparallel feature of these proteins is under positive selection.

**Figure 4:**
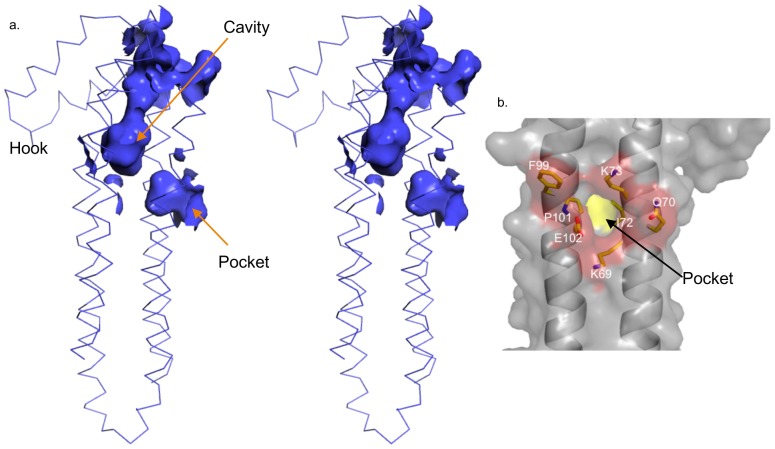
The internal cavity of *Pb*SPECT1Δ41. **a**. Stereoview of the interior cavity in *Pb*SPECT1Δ41 depicted as a surface, and the backbone of *Pb*SPECT1Δ41 as sticks. The positions of the cavity, pocket, and hook are indicated. **b**. A molecular surface representation of *Pb*SPECT1Δ41 with its deep pocket is shown. Residues at the entrance of the pocket are indicated.

Since the presence of parallel/antiparallel helices in four-helix bundles can be a sign of poor side chain packing, we examined the core of *Pb*SPECT1Δ41 [25]. The portion of the structure distal to the ‘hook’ has a close-packed interior, which is composed of a large number of conserved leucines and isoleucines (Fig. 4a). In contrast, a striking and spacious cavity occurs in the interior of the structure proximal to the “hook” (Fig. 4a). The volume of this cavity is ∼750 Å^3^, but in actuality may be just somewhat smaller as six residues in the α1-α2 loop, which is at the top of the cavity, were not modeled. This cavity is lined predominantly by hydrophobic residues (L75, F76, I79, A81, S82, D89, V90, V91, L94, Y97, N156, K160, I163, I164, Y167, G168, K169, V207, L210, I214, I217, Y218, F221), with most of these being conserved in *Pb*SPECT1 homologs (Fig. 2c). Of further note, this cavity is juxtaposed against an unusually deep pocket (145 Å^2^ in area) on the surface of *Pb*SPECT1Δ41 (Fig. 4). Most of the residues lining this deep pocket (K69, Q70, I72, K73, F76, S98, P101, E102, and L105) are also conserved in *Pb*SPECT1 homologs (Fig. 2c). The cavity and the pocket are not continuous, but with motions in the protein, it is possible that the cavity would have access to solvent through the pocket. A second possibility for solvent access to the cavity is near the “hook”. As noted above, this area contains the disordered (and unmodeled) α1-α2 loop residues, and so it is difficult to say based on the present data whether the cavity opens to solvent at this location.

## Conclusions

The structure of *Pb*SPECT1 supplies some clues as to how this protein may function in pore formation and consequent host cell traversal. While the four-helix bundle fold of *Pb*SPECT1 is quite widespread, the parallel/antiparallel alignment of the four helices in *Pb*SPECT1 is quite rare [30], [31]. The great majority of four-helix bundle proteins have their helices tilted with respect to another, which enables favorable packing of side chain packs. One four-helix bundle protein, cytochrome *b_562_*, has been studied in both tilted and parallel/antiparallel conformations. In its heme-bound form, cytochrome *b_562_* (i.e., the holoprotein) was found to have its helices tilted at the canonical angle of ∼25° [32]. Holocytochrome *b_562_* was observed to be stable [33]. However, without the heme bound, cytochrome *b_562_* (i.e., the apoprotein) was found to be only marginally stable and have a poorly packed set of parallel/antiparallel helices [33], [34]. These observations bring up the possibility that the four-helix bundle in *Pb*SPECT1Δ41 is also marginally stable and, correspondingly, conformationally labile. This would accord with a mechanism in which *Pb*SPECT1 is triggered to undergo a conformational change from soluble to membrane-associated or inserted form.

The most distinctive structural feature of *Pb*SPECT1Δ41 is its large interior cavity. It is intriguing to note that the marginally stable apocytochrome *b_562_* has a similar feature. In cytochrome *b_562_*, the hydrophobic residues that contact the heme in the stable holoprotein form become exposed to solvent and form a large exterior cavern in the unstable apoprotein form. The presence of the cavity in *Pb*SPECT1Δ41 further suggests marginal stability, as the interiors of well folded, stable proteins are as densely packed as crystals of small organic molecules [35]. In several cases, the filling of cavities with larger side chains has been found to increase stability [36], [37]. The cavity in *Pb*SPECT1Δ41 may be indicative of a structural defect, but may also constitute a ligand-binding pocket. In this latter regard, it is notable that the residues that line the cavity are mostly hydrophobic and conserved among SPECT1 homologs. As a point of reference for the size of ligands that could be accommodated by the ∼750 Å^3^ cavity of *Pb*SPECT1Δ41, we point out that cholesterol has been measured to occupy a volume of ∼630 Å^3^ [38]. It is tempting to suggest that the binding of a specific ligand, which could be another protein, triggers a conformational change required for membrane activity in *Pb*SPECT1Δ41. The structure of *Pb*SPECT1Δ41 lays the foundation for testing this and other possibilities.

## Supporting Information

Figure S1 **Multiangle light scattering.** Multiangle static light scattering of the *Pb*SPECTΔ41 monomer (red) and dimer (blue) fractions applied to a gel filtration column. The fractions were run separately, but the chromatograms were superimposed for display purposes.
(TIF)

Figure S2 **Electron Density.** Electron density (contoured at 1σ) calculated from a 2Fo-Fc omit map shown as a mesh. The residues shown in the figure, which line the internal cavity of *Pb*SPECTΔ41, were omitted from calculations.
(TIF)

Figure S3 **“V”-shaped dimer.** **a**. Molecular surface representation of the “V”-shaped interaction between two *Pb*SPECT1Δ41 molecules (red and blue). Helices α1 and α2 form the greater part of the interface, with a few residues from α4 being involved as well. **b**. Contents of the *Pb*SPECT1Δ41 crystal unit cell. *Pb*SPECT1Δ41 is shown in ribbon representation, with the four molecules that constitute an asymmetric unit having the same color. There are four asymmetric units in the unit cell.
(TIF)

Figure S4 **State of** ***Pb*** **SPECT1Δ41 in crystals.** Crystallization drops containing crystals of *Pb*SPECT1Δ41 were solubilized in SDS-PAGE buffer and analyzed by Coomassie-stained SDS-PAGE. Lane 1: One-third of a *Pb*SPECT1Δ41 crystallization drop. Lane 2: Two-thirds of a *Pb*SPECT1Δ41 crystallization drop. Lane 3: *Pb*SPECT1Δ41 (∼4 µg). Lane 4: *Pb*SPECT1Δ41 (∼4 µg) diluted 1∶1 with crystallization reservoir buffer. Lane 5: *Pb*SPECT1Δ41 (∼8 µg) diluted 1∶1 with crystallization reservoir buffer. Molecular mass standards are shown between lanes 3 and 4. The crystallization reservoir contains 25% PEG 3350, which results in *Pb*SPECT1Δ41 migrating slightly faster in the crystallization drop (lanes 1 and 2) and in samples containing PEG (lanes 4 and 5) than in the sample containing no PEG (lane 3). No proteolysis of *Pb*SPECT1Δ41 in the crystallization drop was evident.
(TIF)

Table S1 **Sequences of Primers.**
(DOC)
