# Supplementary figures and images for: Structure of the Essential Plasmodium Host Cell Traversal Protein SPECT1
Source: PLoS One. 2014 Dec 5;9(12):e114685. doi: 10.1371/journal.pone.0114685 (PMC4257719; doi:10.1371/journal.pone.0114685)

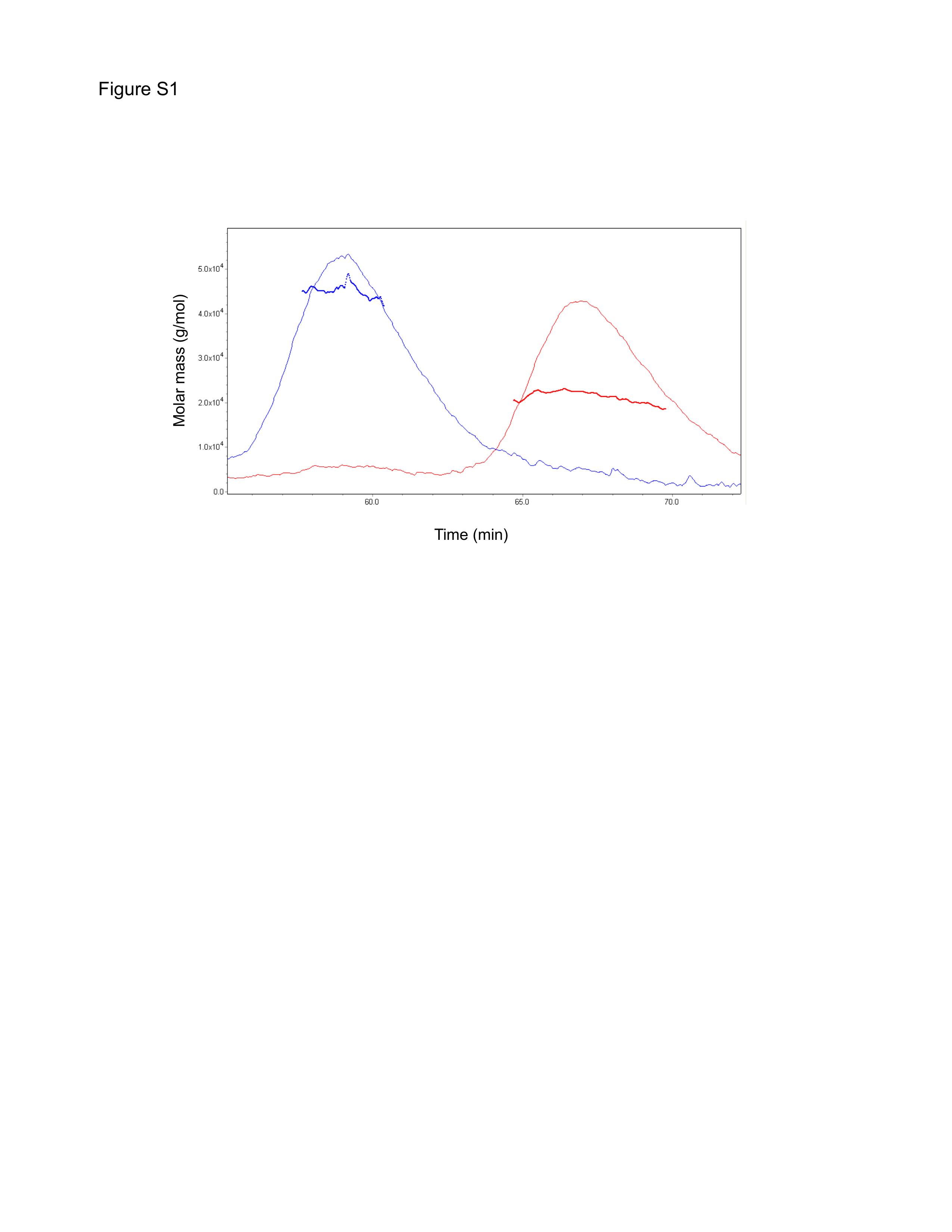

Supplement: Figure S1 — Multiangle light scattering. Multiangle static light scattering of the PbSPECTΔ41 monomer (red) and dimer (blue) fractions applied to a gel filtration column. The fractions were run separately, but the chromatograms were superimposed for display purposes. (TIF) [file pone.0114685.s001.tif]

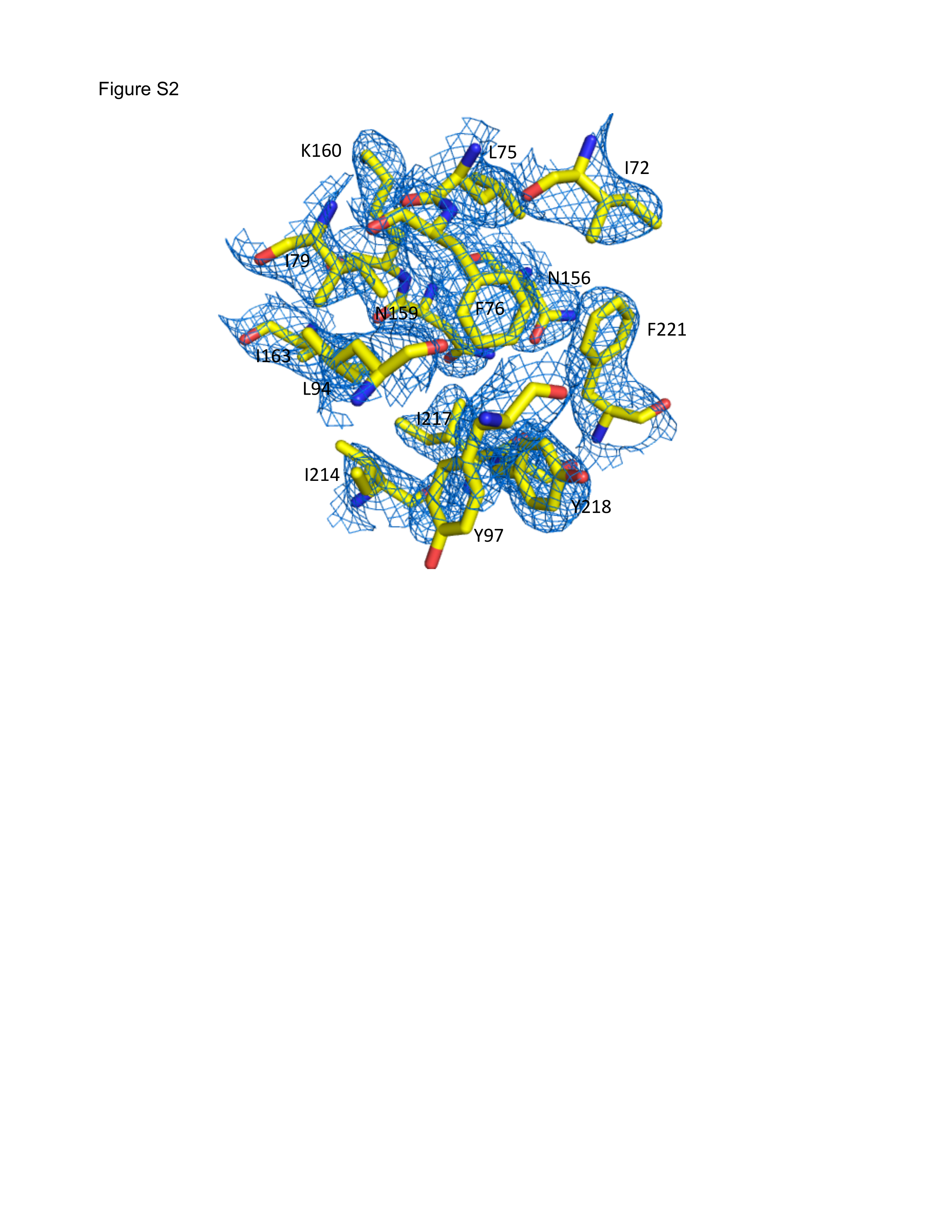

Supplement: Figure S2 — Electron Density. Electron density (contoured at 1σ) calculated from a 2Fo-Fc omit map shown as a mesh. The residues shown in the figure, which line the internal cavity of PbSPECTΔ41, were omitted from calculations. (TIF) [file pone.0114685.s002.tif]

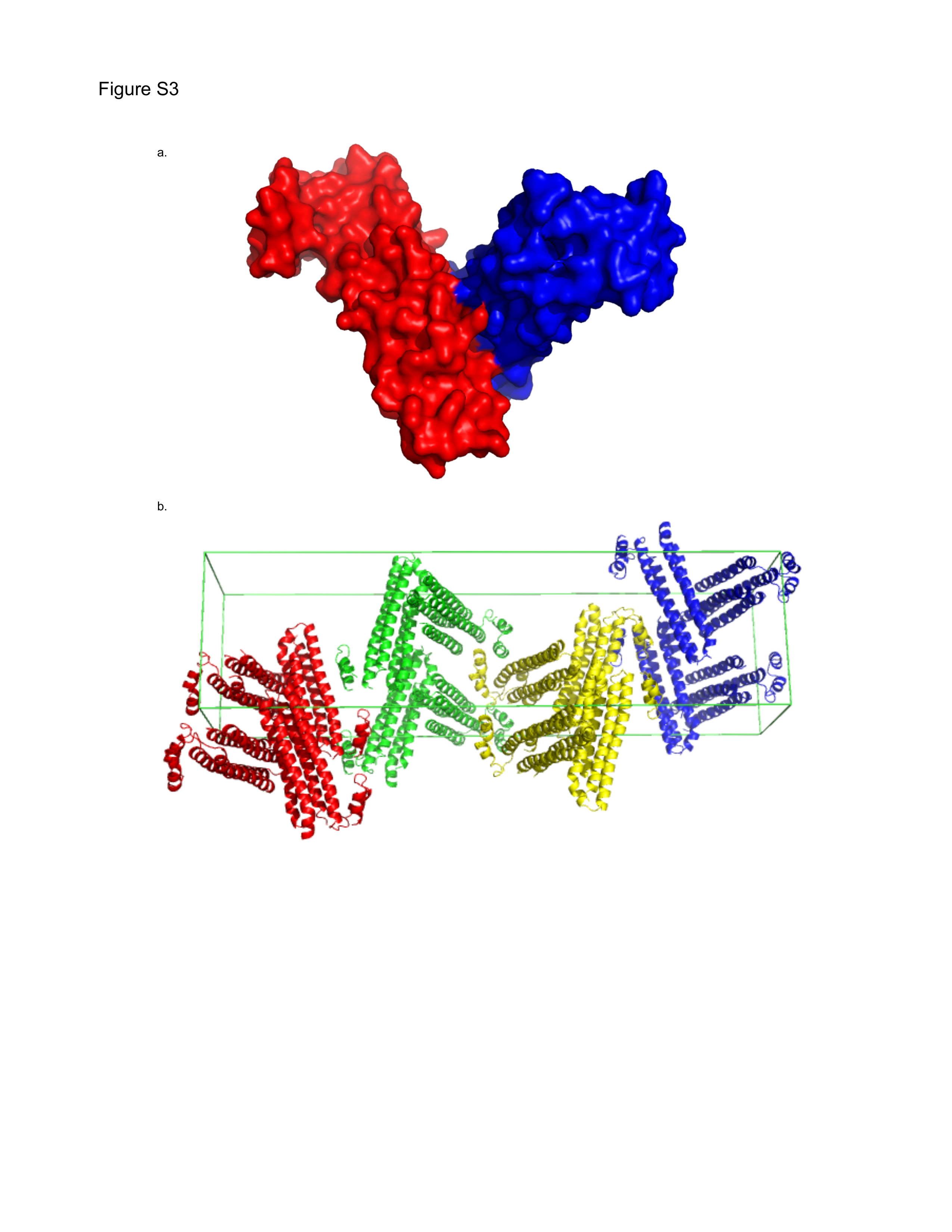

Supplement: Figure S3 — “V”-shaped dimer. a. Molecular surface representation of the “V”-shaped interaction between two PbSPECT1Δ41 molecules (red and blue). Helices α1 and α2 form the greater part of the interface, with a few residues from α4 being involved as well. b. Contents of the PbSPECT1Δ41 crystal unit cell. PbSPECT1Δ41 is shown in ribbon representation, with the four molecules that constitute an asymmetric unit having the same color. There are four asymmetric units in the unit cell. (TIF) [file pone.0114685.s003.tif]

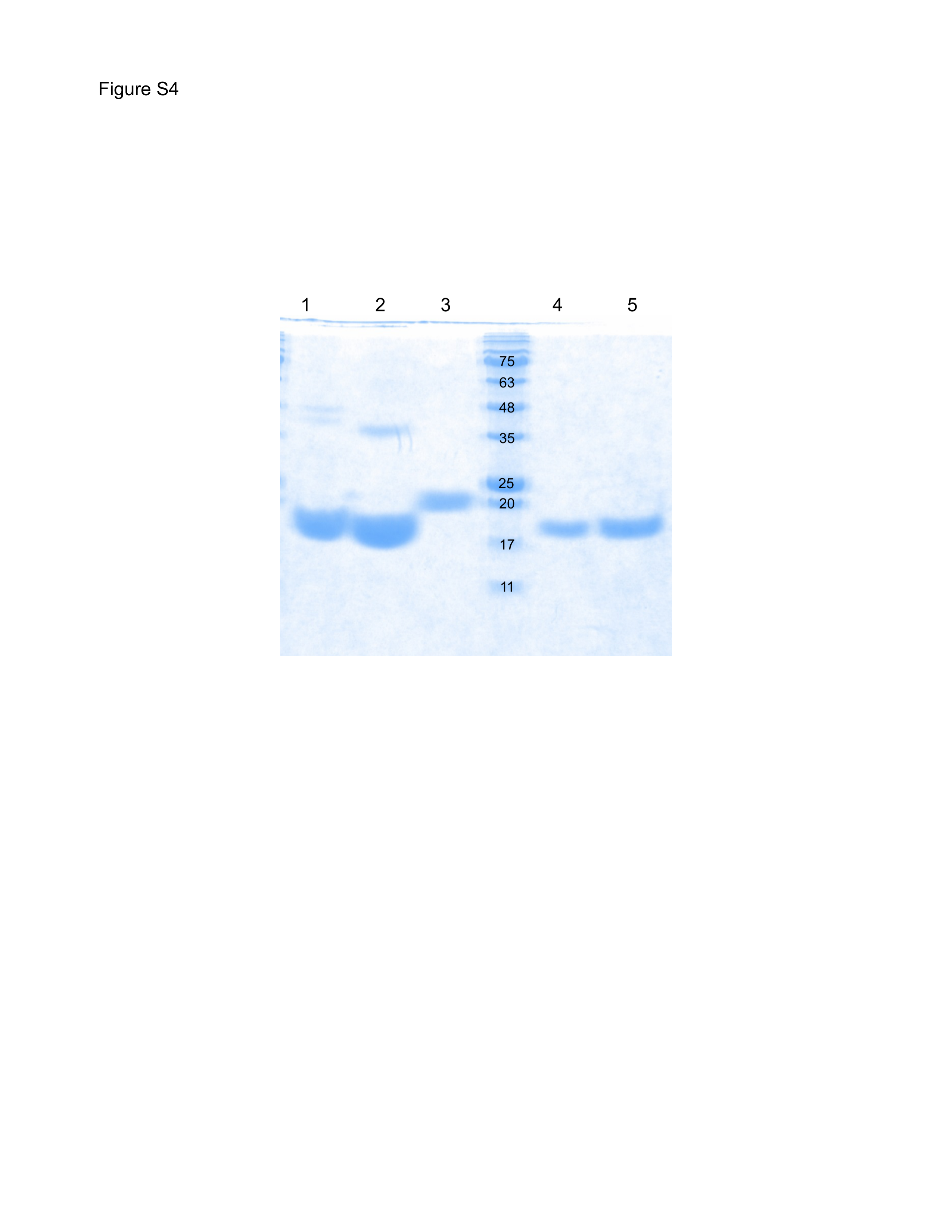

Supplement: Figure S4 — State of Pb SPECT1Δ41 in crystals. Crystallization drops containing crystals of PbSPECT1Δ41 were solubilized in SDS-PAGE buffer and analyzed by Coomassie-stained SDS-PAGE. Lane 1: One-third of a PbSPECT1Δ41 crystallization drop. Lane 2: Two-thirds of a PbSPECT1Δ41 crystallization drop. Lane 3: PbSPECT1Δ41 (∼4 µg). Lane 4: PbSPECT1Δ41 (∼4 µg) diluted 1∶1 with crystallization reservoir buffer. Lane 5: PbSPECT1Δ41 (∼8 µg) diluted 1∶1 with crystallization reservoir buffer. Molecular mass standards are shown between lanes 3 and 4. The crystallization reservoir contains 25% PEG 3350, which results in PbSPECT1Δ41 migrating slightly faster in the crystallization drop (lanes 1 and 2) and in samples containing PEG (lanes 4 and 5) than in the sample containing no PEG (lane 3). No proteolysis of PbSPECT1Δ41 in the crystallization drop was evident. (TIF) [file pone.0114685.s004.tif]
