# Supplementary material for: Structure of the Essential Plasmodium Host Cell Traversal Protein SPECT1
Source: PLoS One. 2014 Dec 5;9(12):e114685. doi: 10.1371/journal.pone.0114685 (PMC4257719; doi:10.1371/journal.pone.0114685)
Supplement: Table S1 — Sequences of Primers. (DOC) [file pone.0114685.s005.doc]

| Forward *Pb*SPECT1Δ24 | gcgcggatccgaaccaaaaggaaataatatctcttta |
| --- | --- |
| Reverse *Pb*SPECT1Δ24 | gcgcctcgagttattttgaaatattctttagtttaatttg |
| Forward *Pb*SPECT1Δ41 | cgcgggatccAATATTGATCATTCGAAAAATAATATCATTGAAGAATTTGACAAACTTTC |
| Reverse *Pb*SPECT1Δ41 | CACACTCGAGTTATTTTGAAATATTCTTTAGTTTAATTTGTAAATCATTAATTAAATCAG |
| L57M | tatatcattactaaagtcatctgacattttgtcaaattcttcaatgatattatttttcgaatgatcaatattag |
| L75M | aacgaagcttctatgtcgagaaacatatcttttatagtttgctttgttgcatttatatca |
| I133M | tacaaacattttttattttttctttttgcatatatatataagcatttgaggaattgataacgtgg |

**Table S1. Sequences of Primers**
